# Postural sway in migraine patients and controls, results from a population based CAMERA-2 study

**DOI:** 10.1186/1129-2377-14-S1-P119

**Published:** 2013-02-21

**Authors:** H Koppen, IH Palm-Meinders, CGC Horlings, GM Terwindt, LJ Launer, MA van Buchem, MC Kruit, MR Bloem, MD Ferrari

**Affiliations:** 1 Hagahospital, Netherlands; 2 Leiden University Medical Center, Netherlands; 3 Radboud University Nijmegen Medical Centre, Netherlands; 4 Laboratory of Epidemiology, Demography and Biometry, USA

## Background

Impairment of balance and oculomotor function have been found in several small clinic based studies investigating migraineurs inter-ictally. Whether these functional abnormalities are due to migraine specific brainlesions or the migraine phenotype, has never been investigated.

## Purpose

To investigate trunk stability during everyday stance and gait tasks in migraineurs and controls in a population based study and to correlate findings with presence of cerebellar infarcts and infratentorial hyperintense lesions (IHLs) on MRI.

## Methods

Trunk sway in the medio-lateral (roll) and anterior-posterior (pitch) plane was measured using two digital angular velocity transducers attached to the lower back. Subjects completed three different trials. All tests were done interictally and investigators were blinded for all participant characteristics. Outcomes are given as trunksway angles and angular velocity in both the roll and pitch plane.

## Results

A total of 190 subjects participated; 71 migraineurs with aura (MA, 72% women, mean age 58 yr), 53 migraineurs without aura (MO, 72% women, 58 yr) and 53 controls (62% women, 55 yr). As an additional positive control group we also investigated trunk sway in 13 patients with Familial Hemiplegic Migraine with a CACNA1A mutation (FHM1, 69% women, 42 yr). Migraineurs (MA and MO) and controls performed equally in all three trials. Trunksway angles and angular velocities of both the roll and pitch plane were significantly higher in FHM patients, compared to MA, MO, and controls, for the simple trial (p=< 0.001.) The difficult trial was completed by none of the FHM patients. Subjects with a cerebellar infarct (n=3) had higher pitch velocity (M=46.8 SD 7.6) while completing the difficult trial compared to the non-infarct group (n=72, M=35.2 SD 9.4), p=0.04. The presence of IHLs did not influence trunk sway measurements in any trial.

## Conclusion

FHM1 patients, but not ordinary migraineurs with or without aura, have diminished postural control inter-ictally. IHLs do not affect trunk sway.

